# Global trends in chronic thromboembolic pulmonary hypertension clinical trials and dissemination of results

**DOI:** 10.1177/20458940211059994

**Published:** 2021-11-18

**Authors:** Micheal C. McInnis, Clement T. Chow, Alexandre Boutet, Sebastian Mafeld, John Granton, Karen McRae, Laura Donahoe, Marc de Perrot

**Affiliations:** 1 Department of Medical Imaging, University of Toronto, Toronto, ON, Canada; 2 Division of Respirology, Department of Medicine, University of Toronto, Toronto, ON, Canada; 3 Department of Anesthesia and Pain Management, 33540 Toronto General Hospital, Toronto, ON, Canada; 4 Division of Thoracic Surgery, Department of Surgery, University of Toronto, Toronto, ON, Canada

**Keywords:** registries, pulmonary embolism, pulmonary hypertension, trials

## Abstract

Treatment options for chronic thromboembolic pulmonary hypertension (CTEPH) are rapidly expanding. The purpose of this study is to identify trends in CTEPH clinical trials and the publication of results. We performed a worldwide review of completed and ongoing clinical trials through searching the ClinicalTrials.gov database and the World Health Organization International Clinical Trials Registry Platform for “CTEPH” and related terms. Entries were classified as pharmaceutical/procedural interventions (Group 1), all other clinical trials (Group 2) and patient registries (Group 3). Trial characteristics and national affiliation were recorded. PubMed was searched for related publications. There were 117 clinical trials registry entries after removing duplicates and non-target records. Group 1 comprised 29 pharmaceutical, 15 procedural, and four combined interventions starting in 2005, 2010, and 2016, respectively. Riociguat and balloon pulmonary angioplasty were the most frequent pharmaceutical and procedural interventions, respectively. The proportion of procedural trials increased over time from 0% of those in 2005–2009 to 29% in 2010–2014 and 54% in 2015–2020. There were 56 entries in Group 2 and 13 in Group 3. Japan was the most frequent national affiliation and the most frequent participating country, present in 28% of all trials. The proportion of entries with published results was highest with Group 3 (62%) and lowest with Group 1 (27%). Thirty percent of all publications occurred in 2020. In conclusion, CTEPH clinical trials are increasingly procedural based, with growth largely attributable to Japan and balloon pulmonary angioplasty. Most trials have not published, but results from balloon pulmonary angioplasty clinical trials are anticipated soon.

## Introduction

Chronic thromboembolic pulmonary hypertension (CTEPH) is an underdiagnosed disease with significant morbidity and mortality when left untreated.^1^ It is most commonly the complication of acute pulmonary embolism (PE) and, although estimates of the prevalence vary, the incidence of CTEPH is likely around 3% of acute PE survivors.^2^ The most important risk factor for CTEPH is recurrent venous thromboembolism; however, the underlying mechanisms that determine who will develop this condition have yet to be fully elucidated.^1^

Once identified, the gold standard, and curative, treatment for CTEPH is pulmonary endarterectomy (PEA) and even patients with disease in the distal segmental vasculature can achieve excellent outcomes with low mortality at expert centers.^3,4^ More recently, a minimally invasive approach of balloon pulmonary angioplasty (BPA) has been shown to be an effective treatment for CTEPH in select patients.^5–^^7^

While surgery is the optimal treatment for an increasing proportion of patients, medical therapy has become a mainstay of care in CTEPH in the presence of residual PH after PEA or when surgery is not feasible. Riociguat, a soluble guanylate cyclase (sGC) stimulator, was the first medical therapy approved specifically for CTEPH. It is used in patients who are not eligible for surgery, in some patients pre-operatively (although not approved and not recommended for this indication), or in patients with residual or recurrent pulmonary hypertension following PEA.^8,9^

A review of the current practices demonstrates that there have been substantial advances in the diagnosis and treatment of CTEPH. However, even recent reviews may present a skewed perspective of the sum of prior and ongoing research because it may take many years to publish the results of ongoing or completed clinical trials.^10^ Furthermore, if the results of a clinical trial are perceived to be negative or the trial is terminated early, investigators may face barriers in publishing their work.^11^

In addition to the published literature, analysis of clinical trial databases provides a complementary, forward-facing perspective in evaluating the current state of CTEPH research. Clinical trial registries contain information about not only completed clinical trials but also planned, ongoing, and terminated trials.^12,13^ We hypothesize that an analysis of registries specifically for CTEPH-focused clinical trials could elucidate the direction of current research by identifying trends over time.

## Methods

### Clinical trials database search

We performed a worldwide search of clinical trial databases by accessing the ClinicalTrials.gov registry and the World Health Organization’s International Clinical Trial Registry Platform (ICTRP). The ClinicalTrials.gov registry was created in 1997 and is based in the United States.^14^ The ICTRP accesses multiple registries, including the Japan Primary Registries Network (JPRN) as well as the European Union, Chinese, and Pan African Clinical Trial Registries.^15^ The search terms were “chronic thromboembolic pulmonary hypertension OR CTEPH OR chronic pulmonary embolism OR chronic thromboembolic disease (CTED) OR CTED” and on the search date 15 July 2020. Two readers (CTC and AB) independently reviewed all entries for duplicate, incomplete, and non-target entries.

### Classification of trials

Two readers (CTC and AB) reviewed all entries to classify each as one of three groups. Group 1 trials related to the treatment of CTEPH using either a pharmacologic or procedural intervention. Trials comparing a procedural and pharmaceutical intervention, or their utilization in sequence, were classified as a hybrid type. Therefore, trials in Group 1 were subclassified as pharmacologic, procedural, or hybrid. Group 2 trials were all remaining entries that were not patient registries. Group 3 were patient registries as defined by the database study type field or when the study title or description contained the word “registry.” Disagreements in classification were resolved by a third reader (MM).

For Group 1 trials, the specific pharmacologic therapy or procedural intervention, the presence or absence of randomization, projected enrollment and the trial phase were recorded. Clinical trial phases 0–2 were grouped as early phase and phases 3–4 were grouped as late phase. Reported industry funding was noted, and the recruitment status of the trial was recorded.

For Group 2, entries were grouped according to study aim as those involving the investigation of (a) CTEPH incidence or prevalence, (b) exercise, rehabilitation, or oxygenation, (c) imaging studies, and (d) others. This group offers a perspective on the directions of CTEPH research in terms of topics of focus. However, given their heterogeneity, they are not readily amenable to meaningful comparison using the metrics employed in Group 1.

For Group 3, patient registries were classified as national or international. Actual or projected enrollment numbers were recorded when available.

### Global distribution

National affiliation of the clinical trial was assigned by the institutional affiliation of the principal investigator or, if not listed, the affiliation of the responsible party field. If the responsible party was a pharmaceutical company, then a national affiliation was not attributed. As clinical trials may be multicenter, we also extracted the countries that were involved in each clinical trial using the study locations field. Each country was only counted once even when multiple sites within that country were listed for a specific database entry.

### Identification of peer-reviewed literature

All clinical trial entries were searched for indexed publications on the ClinicalTrials.gov webpage. PubMed was also searched for all clinical trial identifier numbers. Furthermore, PubMed was then searched for the principal investigator of each database entry to identify any additional publications attributable to the clinical trial, and this search was performed 16 July 2020 by one reviewer (CTC). Publications were classified as original research and others (i.e. reviews, letters, protocols, etc.).

### Statistical analysis

Continuous variables were described using mean or median with standard deviation or range where appropriate. For identification of trends over time, clinical trials were grouped by start date. For all entries, the trial start date was recorded and defined as the actual date on which the first participant was enrolled in the clinical study or, if unavailable, the estimated study start date. The estimated study start date was defined as the reported date that the investigators estimated will be the study start date.

## Results

### Database search results

**Fig. 1.**
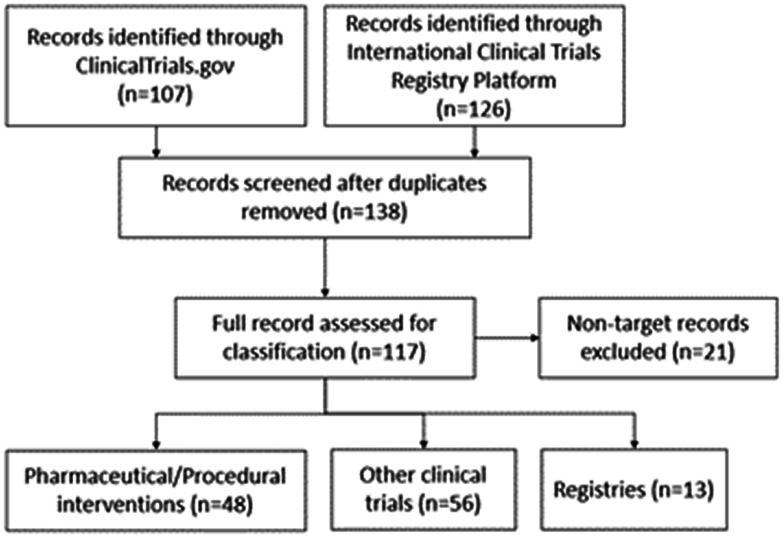
Clinical trial database search results and selection.

There were 107 entries in the ClinicalTrials.gov database and 126 in the ICTRP database searching up to July 2020, with 117 total entries after removing duplicates and non-target entries (Fig. 1). There were 48 Group 1 entries, 56 Group 2 entries, and 13 Group 3 registries. All trials pertained to CTEPH with no additional clinical trials identified for the search terms of “chronic thromboembolic disease” or “CTED.”

**Fig. 2.**
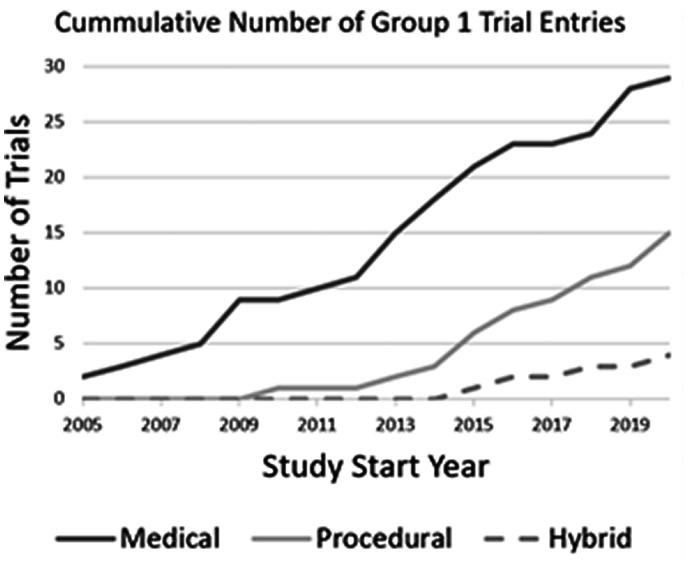
Clinical trials over time for medical, procedural, and hybrid interventions. The first database entry for a procedural intervention was in 2010 and, for a hybrid intervention, in 2016.

The earliest study start date was 29 August 2005, corresponding to the BENEFIT (Bosentan Effects in iNopErable Forms of chronic Thromboembolic pulmonary hypertension) pharmaceutical trial.^16^ The earliest procedural trial start date was 1 July 2010 (NCT01163422), and the earliest hybrid trial was posted in 2016. The first registry captured by the search had a start date in 2009 (NCT01417338) and the first posted registry exclusively for CTEPH patients was the U.S. CTEPH Registry (NCT02429284) in April 2015, acknowledging that the International CTEPH Registry, which enrolled patients between 2007 and 2009, was not present in the clinical trial databases (Fig. 2).^17^

**Fig. 3.**
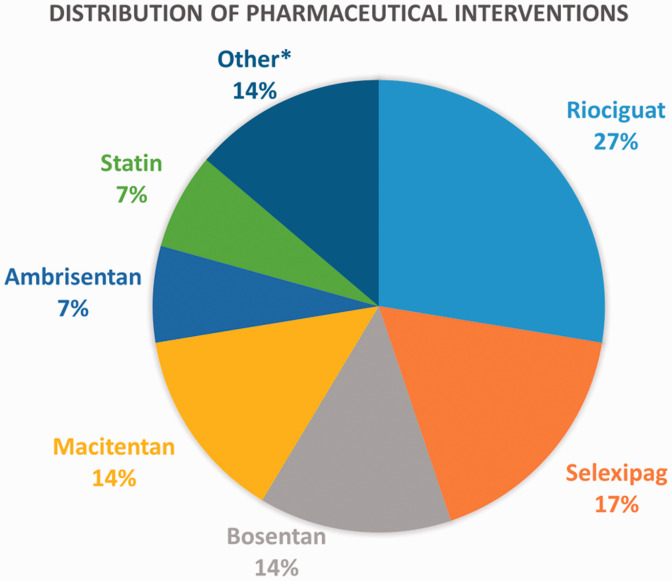
Types of pharmaceutical interventions in Group 1 database entries. *Other pharmaceutical interventions include N-acetyl cysteine (1), edoxaban and warfarin (1), treprostinil sodium (1), and inhaled BAY1237592.

**Fig. 4.**
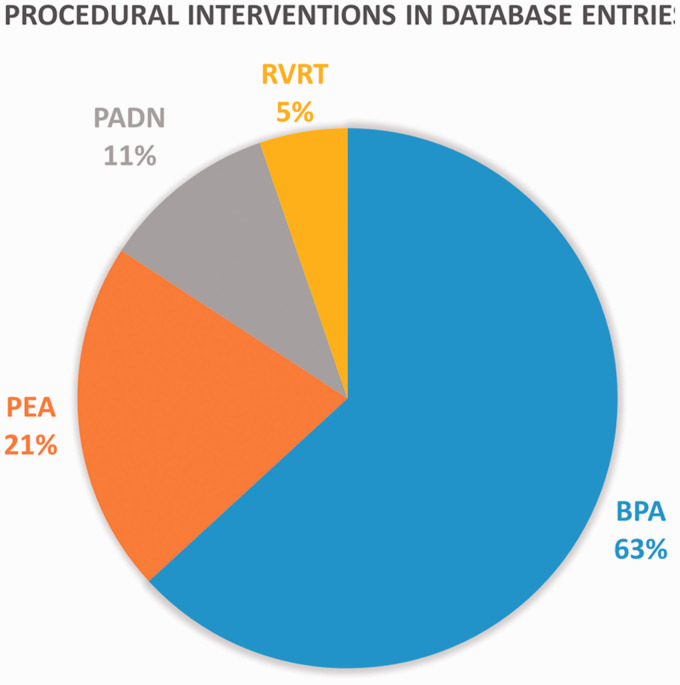
Types of procedural intervention in Group 1 database entries. BPA: balloon pulmonary angioplasty; PEA: pulmonary endarterectomy; PADN: pulmonary artery denervation; RVRT: right ventricular resynchronization therapy.

Of the 48 Group 1 trials, 29 were pharmaceutical, 15 were procedural, and 4 were hybrid interventions. The most frequent pharmaceutical interventions were riociguat (33%) and selexipag (15%) (Fig. 3). Two clinical trial database entries also pertained to the use of simvastatin and atorvastatin in 2007 and 2008, respectively, though the results have not been identified. The most frequent procedures were BPA (63%) and PEA (21%) (Fig. 4). A summary of the identified BPA clinical trials is provided in Table 1.

**Table 1.** Clinical trials involving balloon pulmonary angioplasty ordered by date first posted to the clinical trial database.

Clinical trial name	Clinical Trial Identifier Number	Date first posted	Recruitment status	Randomization	Projected enrollment (n)	National affiliation
Safety and efficacy of BPA for CTEPH	UMIN000017191	1 May 2015	Not recruiting	No	5	Japan
Safety and efficacy of bioabsorbable stent in percutaneous transluminal pulmonary angioplasty	UMIN000018094	1 July 2015	Recruiting	No	20	Japan
Multicenter Randomized controlled trial based on Balloon Pulmonary Angioplasty for chronic thromboembolic pulmonary hypertension (MR BPA)	UMIN000019549Sub studies:UMIN000021466jRCTs031180239	28 October 2015	Recruiting	Yes	60	Japan
Riociguat Versus Balloon Pulmonary Angioplasty in Non-operable Chronic thromboEmbolic Pulmonary Hypertension (RACE)	NCT02634203	17 December 2015	Not recruiting	Yes	124	France
BPA in non-operable CTEPH patients	NCT02964390	16 November 2016	Unknown status	No	50	Poland
A randomized controlled study to evaluate the efficacy and safety of pressure-wire-guided BPA for CTEPH	UMIN000022888	1 December 2016	Not recruiting	Yes	20	Japan
Hemodynamic effects of BPA at rest and during exercise in CTEPH	NCT04052243	9 August	Not recruiting	No	80	Denmark
Safety and efficacy of BPA in China	NCT04206852	20 December 2019	Recruiting	No	200	China
Clinical study of BPA for patients with CTEPH	NCT04326777	30 March 2020	Recruiting	No	27	China
PRACTICE study	ChiCTR2000032403	27 April 2020	Not recruiting	No	60	China

BPA: balloon pulmonary angioplasty; CTEPH: chronic thromboembolic pulmonary hypertension; jRCTs: Japan Registry of Clinical Trials; NCT: National Clinical Trial; PRACTICE: Efficacy and safety of refined balloon pulmonary angioplasty combined with riociguat in the treatment of inoperable chronic thromboembolic pulmonary hypertension: a single center, open label, prospective, optimal, randomized, parallel controlled clinical study; UMIN: University Hospital Medical Information Network.

**Fig. 5.**
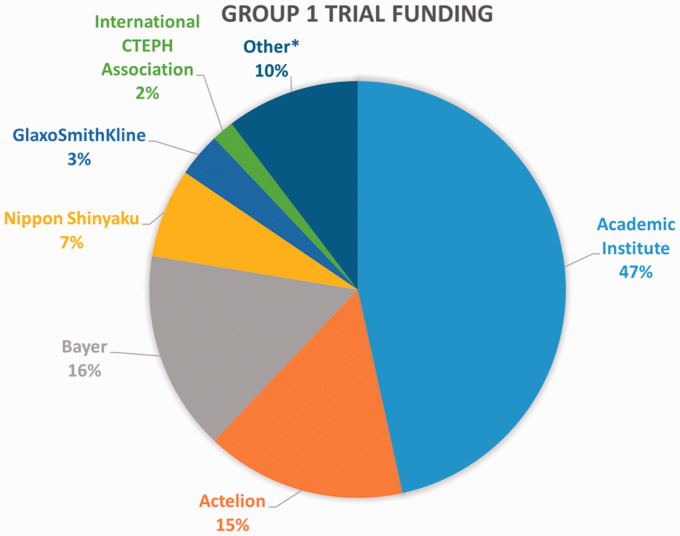
Clinical trial sponsorship in clinical trials of a pharmaceutical or procedural intervention (Group 1). Academic institutes included all hospitals, universities or other institutes and was the most frequent sponsor of Group 1 clinical trials. *Other sponsors with a single occurrence include Medtronic, SciPharm, Mitsubishi, General Laboratory BML, EPS Corporation, and Imepro Inc.

In Group 1, there were 12 (25%) early phase trials and 14 (29%) late phase trials with no trial phase reported in 22 (46%) cases. Half (n = 24) were randomized, with more pharmaceutical trials being randomized than procedural trials (56% vs. 45%, respectively). Projected enrollment for all Group 1 trials was a median of 62 participants (range 2 to 1298 participants). Projected enrollment was similar for pharmaceutical, procedural, and hybrid trials with a median of 72 (range 2–1298), 60 (range 5–200), and 70 (range 60–124) participants, respectively. Nearly half (46%) of Group 1 trials had industry-sponsorship, with Actelion and Bayer as the most frequent industry funder of nine clinical trials each (Fig. 5). Industry funding was more common in pharmaceutical trials than procedural trials (76% vs. 7%, respectively).

The proportion of procedural to pharmaceutical trials increased from 0% in 2005–2009 to 29% in 2010–2014 and 54% in 2015–2020, with all four hybrid entries occurring after 2015. The proportion of Group 1 trials registered as completed was 21% and decreased over time from 36% in 2005–2009 to 22% in 2010–2014 and 3% in 2015–2020. Conversely, there was a greater proportion of Group 1 trials registered as recruiting over time from 7% in 2005–2009, to 11% in 2010–2014 and 42% in 2015–2020.

Early termination was reported in three clinical trials due to (1) a reported change in development strategy for a study of macitentan (NCT03809650), (2) slow patient recruitment for a study of bosentan after macitentan became available (NCT02970851), and (3) initially not specified for the PEA Bridging study (NCT03273257) but, at the time of writing, listed as slower than expected recruitment in addition to limitations imposed by the COVID-19 pandemic.

Group 2 was heterogeneous with the largest proportion related to exercise, oxygenation, or ventilation (n = 19, 34%) followed by clinical trials investigating CTEPH incidence (32%), imaging-based studies (18%) and others (16%) including a broad range of topics from the study of matrix metalloproteinase to the interrogation of right ventricular myocardial biopsies.

There were 13 patient registries. Actual patient enrollment was available in four of the registries, the largest being 1019 participants in the International CTEPH Association’s New International CTEPH Database (NCT02656238), with the United States CTEPH Registry reporting 754 participants (NCT02429284), the International BPA Registry reporting 502 participants (NCT03245268), and the EMEA (Europe/Middle East/Africa) CTEPH Registry reporting 231 participants (NCT02637050).

### Global distribution

**Fig. 6.**
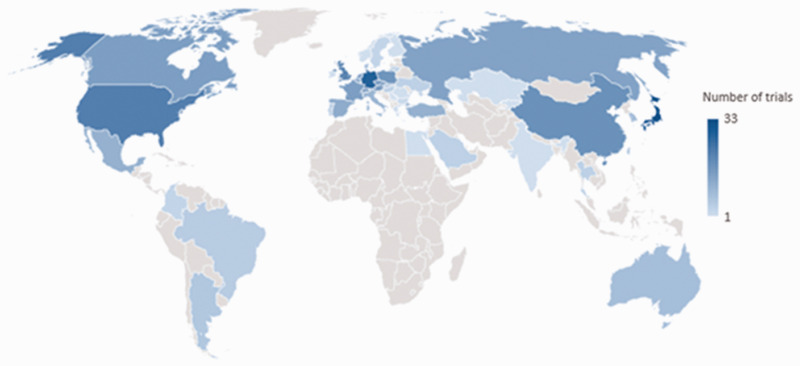
Global distribution of clinical trials map demonstrating clinical trial database entry participation. CTEPH: chronic thromboembolic pulmonary hypertension.

Of the total 117 database entries, 95 were attributable to a country through a principal investigator or responsible party affiliation. The most frequent national affiliation was Japan (n = 18) followed by China (n = 11) and the United States (n = 10) (Fig. 6). There were 21 clinical trial entries attributable only to a pharmaceutical company and these were most frequently Bayer (n = 9) and Actelion (n = 6). There was one entry attributed to the International CTEPH Association. Pharmaceutical trials were most frequently attributed to a pharmaceutical company (Bayer (n = 7) and Actelion (n = 6)), and procedural trials were most frequently nationally affiliated (Japan (n = 5) and China (n = 4)).

Because 18% of database entries were attributable to a multinational pharmaceutical company, we additionally evaluated participation in clinical trials as indicated by the study location field. By this analysis, Japan again had the most frequent participation, involved in 28% of all clinical trial database entries, followed by Germany (24%), the United States (19%), and China (15%). Twenty-two of the clinical trials were multinational with participation ranging from 2 to 32 countries and the most diverse being NCT03689244.

### Literature search

**Fig. 7.**
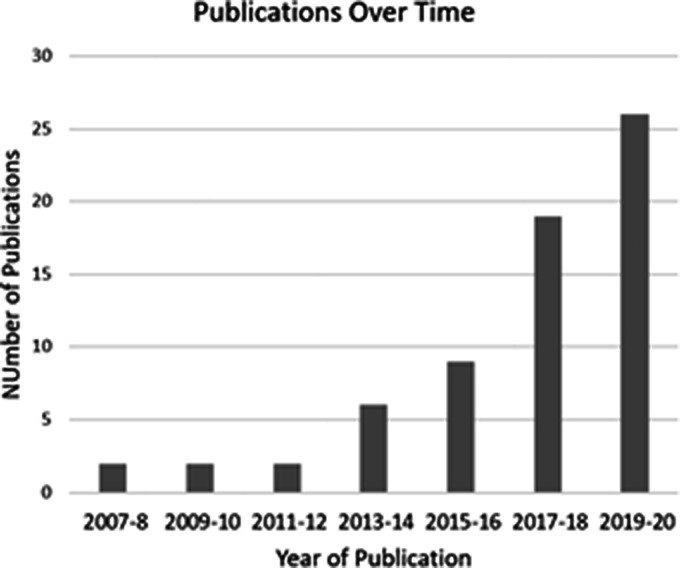
Chart of all publications for all clinical trial database entries over time grouped by two-year intervals.

There were 66 publications identified searching all clinical trial identifiers up to 31 December 2020, including 46 articles of original research attributable to 37 unique database entries. The proportion of database entries with published original research was highest in Group 3 (54%) compared to 34% in Group 2 and 21% in Group 1. The remaining publications indexed by our search consisted of six reviews, five abstracts, five protocols, one meta-analysis, one commentary, one clinical practice guideline, one task force report, and one graduate thesis. There was an increasing number of publications over time, with 30% of publications being in 2020 (Fig. 7).

## Discussion

Clinical trials in CTEPH are diverse but substantially focused on pharmaceutical and procedural interventions. Clinical trials involving a pharmaceutical intervention were most often industry-funded, multinational and evaluating riociguat, whereas procedural trials were infrequently industry-funded, were more frequently based in Japan or China, and most frequently evaluated BPA. We found an increasing proportion of recruiting clinical trials over time across all groups with a trend shifting from pharmaceutical, to procedural and, most recently, hybrid approaches. Globally, Japan was affiliated with the largest number of clinical trials and had the highest general participation. Although we have identified more than one hundred clinical trial database entries, only a minority have published their results.

We found an increasing proportion of procedural clinical trials over time largely attributable to BPA and predominantly from Japan and China. The global distribution of clinical trials is not only of geographic interest, as there are important differences in the Japanese and European CTEPH cohorts both in patient characteristics and disease phenotype.^18^ The use of BPA in Europe is increasing but is being reported primarily in retrospective studies with few entries identified in the clinical trial databases.^19–^^21^ (Table 1). In addition to BPA, our search identified pulmonary artery denervation as the subject of two clinical trials based in Russia and China, with results having been recently published by Romanov et al.^22^

Around one in four patients will have residual pulmonary hypertension after PEA and it remains unclear how best to integrate medical therapy or BPA.^9^ We identified four hybrid clinical trials focused on comparing treatments. The PEA Bridging trial, comparing PEA to PEA and pre-operative riociguat, was terminated early, in part related to the COVID-19 pandemic (NCT03273257). The multicenter randomized controlled trial based on BPA for CTEPH (MR BPA study) that compares BPA to medical management is yet to publish its results (UMIN000019549).^23^ We also identified a randomized control trial comparing BPA and riociguat to riociguat alone in the Chinese Clinical Trial Registry (ChiCTR2000032403), but this is not yet reported as recruiting. Finally, Riociguat Versus BPA in Non-operable Chronic thromboembolic Pulmonary Hypertension (RACE) trial comparing medical therapy to BPA has been completed, and the publication of results is expected soon (NCT02634203).

Only a minority of clinical trial database entries have published their study results. The publications that we did identify were largely based on early multicenter pharmaceutical trials, such as the clinical trials for bosentan and riociguat in CTEPH.^8,16^ Although most procedural trial entries have not published results, we found exponential growth in the number of publications, with 30% of clinical trial publications having occurred in 2020 alone. While this trend is expected to continue, the full impact of the COVID-19 pandemic on the completion of ongoing clinical trials is yet to be seen.^24^

There are several limitations to this analysis. Firstly, we did not identify clinical trials prior to 2005 despite the ClinicalTrials.gov registry beginning in 1996 and the ICTRP in 2005. For example, the long-term use of sildenafil in inoperable CTEPH clinical trial that started enrolling in 2004, was registered with the UK National Research Register database but was not captured by the ICTRP.^25^ The second limitation is that, although features such as phase and randomization were extracted, we could not perform a more thorough assessment of the quality of the clinical trials and we could not assess the accuracy of the data reported. Finally, although there may be a clinical trial database entry with a proposed start date, it is possible that some clinical trials will never be performed or completed.

Overall, we found that CTEPH clinical trials are increasingly procedural based with growth largely attributable to Japan and the investigation of BPA. Most trials have not published, but results from BPA clinical trials are anticipated soon.
